# Exploring the Healthcare Experiences and Preferences of LGBT+ People: An Online Asynchronous Focus Group Study

**DOI:** 10.1111/jan.70210

**Published:** 2025-09-03

**Authors:** John P. Gilmore, David J. Field

**Affiliations:** ^1^ School of Nursing, Midwifery and Health Systems University College Dublin Dublin Ireland; ^2^ Gay Men's Health Service Health Service Executive Dublin Ireland; ^3^ Department of Applied Health Science University of Aberdeen Aberdeen UK

**Keywords:** gender, health equity, LGBT, mental health, nursing, sexual health, sexuality

## Abstract

**Aim:**

To explore experiences of LGBT+ individuals in accessing and receiving healthcare in Ireland, and to identify opportunities for more inclusive and equitable healthcare delivery.

**Design:**

A descriptive qualitative study using asynchronous online focus groups.

**Methods:**

Asynchronous online focus groups were conducted using the MURAL collaborative platform over a three‐week period in 2023. Participants (*n* = 43) self‐selected into one of three focus groups based on gender and sexual identity. Data were analysed using reflexive thematic analysis to generate key themes.

**Results:**

Four themes were identified: (1) Culturally aware healthcare professionals; (2) Access and information; (3) Specialist versus universal services; and (4) Mental health support. Participants reported frequent experiences of misgendering, heteronormative assumptions, and provider inexperience. Geographic inequities, unclear referral pathways, and a lack of centralised, inclusive information were also key barriers. While specialist services were valued, participants advocated for a dual approach that integrates LGBT+ competence into all healthcare settings.

**Conclusion:**

Despite legal and social progress, LGBT+ individuals in Ireland continue to encounter significant barriers to equitable healthcare. The findings highlight a need for improved cultural competence, better access to inclusive services, and systemic reform.

**Implications for the Profession and/or Patient Care:**

Healthcare providers must receive comprehensive training in LGBT+ health to ensure respectful, appropriate, and inclusive care. Enhanced visibility of inclusive providers and improved service pathways are needed to address current inequities, particularly in mental health and gender‐affirming care.

**Impact:**

This study identifies key gaps in provider competence, service accessibility, and mental health provision. Findings will inform healthcare education, policy development, and service design to improve experiences and outcomes for LGBT+ individuals in both urban and rural contexts.

**Reporting Method:**

This study adhered to the Standards for Reporting Qualitative Research (SRQR).

**Patient or Public Contribution:**

No patient or public contribution was involved.

## 1 Introduction

Healthcare delivery to lesbian, gay, bisexual, and transgender (LGBT) communities is increasingly recognised as an area requiring urgent attention, as mounting evidence highlights significant health inequities experienced by these populations (Higgins et al. 2016; Hughes et al. 2023; Matthews et al. 2018; Medina‐Martínez et al. 2021; Mulé 2015; Reisner et al. 2016; Zeeman et al. 2019). While strides in legal protections and growing social acceptance have been made in many countries, systemic challenges in healthcare delivery remain. Discrimination, social exclusion, and a lack of cultural competence among healthcare providers continue to negatively impact the quality of care received by LGBT+ individuals, creating persistent barriers to health equity (Fish et al. 2021). These disparities not only undermine the physical and mental well‐being of LGBT+ communities but also present broader challenges for healthcare systems striving for inclusivity and equitable access.

Although the international evidence base around LGBT+ health has grown, research specific to healthcare delivery for LGBT+ communities remains limited, particularly in Ireland (Higgins et al. 2016). Studies consistently demonstrate that LGBT+ populations face elevated rates of mental health issues, such as depression, anxiety, and suicidal ideation, often exacerbated by experiences of minority stress and discrimination (Higgins et al. 2016; Bryan and Mayock 2017; Cullinan et al. 2024; de Vries et al. 2023; Higgins et al. 2024). Other disparities noted internationally include higher rates of substance use disorders and unique physical health risks, such as increased vulnerability to HIV and sexually transmitted infections (STIs) among gay and bisexual men, as well as significant challenges faced by transgender individuals in accessing gender‐affirming care (Zeeman et al. 2019; Reisner et al. 2016).

While legislative milestones such as marriage equality and the Gender Recognition Act have improved the legal landscape for LGBT+ individuals in Ireland, these changes have not fully translated into equitable healthcare delivery. Discrimination, assumptions rooted in cis‐heteronormativity, and a lack of provider knowledge about LGBT‐specific health needs remain pervasive barriers in healthcare settings (Gilmore 2023). Addressing these gaps in research and practice is essential to improving healthcare delivery for LGBT+ communities in Ireland and ensuring that their needs are met within an inclusive and equitable system.

While discrimination and exclusion remain key barriers in healthcare, these experiences are often rooted in heteronormative and cisnormative assumptions that shape service structures, provider training, and patient interactions (Zeeman et al. 2019; Fish et al. 2021). International evidence from the UK and other European contexts shows how these normative frameworks result in providers defaulting to assumptions about patients' gender identity, sexual orientation, and family structures, often rendering LGBT+ individuals invisible or ‘othered’ within care systems (Hudson‐Sharp and Metcalf 2016; Yoursky et al. 2018). Such environments not only contribute to poorer mental and physical health outcomes, but also deter individuals from seeking care due to anticipated stigma or lack of appropriate knowledge among providers (Alencar Albuquerque et al. 2016). Understanding the persistence of these structures is critical for informing inclusive healthcare design.

The aim of this study was to explore the experiences of LGBT+ individuals in accessing and receiving healthcare in Ireland. Through focus group discussions guided by four open‐ended prompts, the study sought to understand participants' perspectives on their healthcare experiences, what they value in healthcare provision, opportunities for improving services, and any additional concerns or insights related to LGBT+ healthcare in the Irish context.

## 2 Methods

### 2.1 Study Design and Theoretical Underpinning

This study employed a descriptive qualitative design, informed by a social constructionist epistemology. Social constructionism posits that individual experiences and understandings, particularly regarding health and healthcare, are actively shaped through social interactions, cultural contexts, and linguistic constructs (Gergen 2009). Within this epistemological framework, knowledge is not viewed as objective or fixed, but rather as context‐dependent and co‐created through dialogue and social processes (Burr 2015).

Aligned with this theoretical approach, we selected Braun and Clarke's reflexive thematic analysis (Braun and Clarke 2006, 2022), which emphasises the active role of researchers in identifying, interpreting, and constructing meaning from qualitative data. This analytical method was particularly suited to exploring the nuanced and socially constructed nature of LGBT+ individuals' experiences with healthcare, allowing us to foreground participants' subjective interpretations and meanings within their specific socio‐cultural contexts.

### 2.2 Asynchronous Online Focus Groups

This descriptive qualitative study used online asynchronous focus groups to explore the healthcare preferences and experiences of LGBT+ communities in Ireland. Asynchronous online focus groups offer an innovative qualitative data collection method that is gaining widespread utility and resonance in our increasingly digital and interconnected social world; modifying traditional in‐person qualitative focus group methods for use in online settings (Rivaz et al. 2019). Unlike traditional face‐to‐face or synchronous virtual focus groups, asynchronous formats provide participants with greater flexibility to engage on their own schedules, eliminating time constraints and reducing the logistical challenges often associated with group‐based qualitative research. This approach is particularly valuable for reaching diverse populations, including those who may face barriers to participation in traditional research settings, such as individuals living in rural or remote areas, those with disabilities, or those who require anonymity due to the sensitive nature of the topic being studied (Gordon et al. 2021).

By allowing participants to reflect on prompts and respond at their own pace, asynchronous online focus groups can yield deeper, more considered insights, enabling researchers to capture rich, nuanced data. Furthermore, the use of collaborative platforms fosters dynamic interaction among participants, encouraging the exchange of ideas and perspectives while maintaining the anonymity that can be crucial for sensitive topics, such as healthcare experiences within marginalised communities.

The MURAL online collaborative platform (MURAL n.d.) was used to facilitate the focus groups. It is a digital tool that enables participants to contribute ideas in real time or asynchronously using virtual ‘sticky notes’ on a shared board. In this study, we used MURAL as a data collection method to allow for flexible and anonymous participation, an approach we felt was particularly appropriate given the sensitive nature of the topic and the diversity of participants' experiences. MURAL is a commercially available tool that we adapted for qualitative research purposes. Participants engaged anonymously and asynchronously over a three‐week period in February and March 2023. Ethical approval for the study was granted by the UCD Human Ethics Research Committee, reference LS‐21‐77, and informed consent was obtained electronically through Google Forms.

### 2.3 Participants

A total of 50 participants provided informed consent, with 43 actively contributing to the focus groups by posting at least one response on the MURAL. Participants were asked to self‐identify their gender, age, ethnicity, place of residence, disability status, and income level. Of the participants, 54% (*n* = 27) identified as men, women accounted for 26% (*n* = 13) of participants, non‐binary individuals made up 16% (*n* = 8), and 2% (*n* = 1) identified as agender. Participants ranged in age, with 22% (*n* = 11) aged 18–25, 38% (*n* = 19) aged 26–35, 20% (*n* = 10) aged 36–45, 16% (*n* = 8) aged 46–55, and 4% (*n* = 2) aged 56–65.

Most participants (84%, *n* = 42) were born in Ireland, while 16% (*n* = 8) were born outside the country, representing diverse origins, including the UK, Germany, USA, Brazil, Canada, and Belarus. 44% of participants (*n* = 22) lived in Dublin, 28% (*n* = 14) resided in rural areas, and 22% (*n* = 11) lived in other urban areas. All demographic information is contained in Table 1.

**TABLE 1:** Demographic information.

Category	Percentage	Count (*n*)
Gender identity		
Cisgender men	40	20
Transgender men	12	6
Cisgender women	8	4
Transgender women	18	9
Non‐binary	16	8
Agender	2	1
Age		
18–25	22	11
26–35	38	19
36–45	20	10
46–55	16	8
56–65	4	2
Birthplace		
Born in Ireland	84	42
Born outside Ireland	16	8
Ethnicity		
White Irish	84	42
Other White	12	6
Black	4	2
Asian	2	1
Mixed‐race	2	1
Residency		
Dublin	44	22
Rural areas	28	14
Other urban areas	22	11
Not specified	6	3
Disability status		
With disability	16	8
Without disability	78	39
Prefer not to disclose	6	3
Income (yearly €)		
0–€9999	12	6
€10,000–€39,999	32	16
€40,000–€99,999	46	23
Above €100,000	8	4
Prefer not to disclose	6	3

### 2.4 Data Collection

Participants self‐selected one of three focus groups based on their identities and experiences: (1) gay, bisexual, and men who have sex with men (GBMSM; *n* = 18), (2) lesbian, bisexual, and women who have sex with women (LBWSW; *n* = 6), and (3) transgender participants (TGD; *n* = 19). Where a participant's identity may have intersected across these groups (e.g., a bisexual trans man), they were able to choose which group they participated in. Each participant chose an anonymous avatar to ensure confidentiality during the focus group discussions. A short demonstration video was embedded into the email inviting participants to contribute.

The focus groups were designed to facilitate inclusive and flexible participation. Each group was hosted on a dedicated MURAL board that remained active for three weeks. Participants could log in at any time, as often as they liked, to contribute to discussions and respond to other participants' inputs. Contributions were made by typing text into a ‘sticky note’ and these notes could be moved around and linked to others' comments. To encourage ongoing engagement, biweekly reminder emails were sent to participants during the live period. While the level of contribution varied, with some participants adding a single sticky note per area and others contributing multiple, each participant engaged with all four prompts, ensuring a broad level of participation across the dataset.

Each focus group included four open‐ended prompts:

1. What has receiving healthcare in Ireland been like for you?

Please share some positive and/or negative experiences of accessing healthcare in Ireland. Let us know what was good or bad about the experience.

2 What's important to you about the healthcare you receive?

Tell us what's most important to you about the healthcare you receive; it could be something like cost or location, or something about the setting or healthcare provider.

3 Opportunities for healthcare provision

Are there any particular areas of healthcare (GP care, Cancer Care, Older persons care, Sexual Health, Mental Health, Gynaecological Care etc.) which you think could be provided specifically to your community in a targeted way?

4 Is there anything else about healthcare for LGBT+ people in Ireland you would like to bring up?

The asynchronous format allowed participants to reflect on their responses and engage meaningfully with others in their group. All data from the MURAL boards were securely downloaded and stored for analysis.

### 2.5 Data Analysis

Data from the MURAL boards were analysed using Braun and Clarke's (2006) reflexive thematic analysis, which involves six clearly defined phases. This process was led by the principal investigator, with all stages of analysis and theme development reviewed and refined in collaboration with the second author to ensure rigour and consistency. The analysis began with familiarisation with the data, during which transcripts of contributions from the MURAL platform were read repeatedly by the research team to gain an in‐depth understanding of participants' accounts and to identify initial ideas and impressions. This was followed by the systematic coding of all data, with codes reflecting significant features relevant to the research questions, such as participants' healthcare experiences, barriers, and suggestions for improvement. Coding was conducted inductively, driven primarily by participants' narratives rather than pre‐existing theories. The codes were then reviewed and organised into broader patterns or potential themes, with initial thematic maps collaboratively developed by the researchers to visually represent these candidate themes. These themes were critically examined and refined iteratively against the original dataset, with themes lacking sufficient evidence or coherence being merged, modified, or discarded to ensure an accurate representation of the data. Through team discussions, each theme was then clearly defined and named to capture its underlying meaning and scope, and key illustrative quotes were selected to exemplify each theme. Finally, the themes were narrated in the results section, incorporating vivid participant quotes to provide compelling accounts of LGBT+ healthcare experiences and preferences, before being integrated with relevant literature and theoretical frameworks in the discussion to contextualise the findings and highlight their implications.

This structured analytical process ensured transparency, rigour, and coherence, providing a nuanced account of participants' healthcare experiences in the Irish context.

### 2.6 Rigour and Researcher Reflexivity

To ensure methodological rigour, we applied Lincoln and Guba's (1985) four criteria for trustworthiness in qualitative research: credibility, dependability, confirmability, and transferability.

- Credibility was enhanced through prolonged engagement with the data and regular peer debriefing within the research team during analysis. We incorporated participants' direct quotes to ensure that findings authentically reflected their experiences. Reflexivity was maintained throughout to minimise the influence of the researcher's assumptions.
- Dependability was supported by maintaining a clear audit trail of analytical decisions, including documentation of coding decisions, theme development, and data interpretation. The consistent use of Braun and Clarke's six‐step framework further ensured procedural coherence.
- Confirmability was addressed through ongoing reflexive practice, with the lead researcher maintaining memos and reflective notes to critically examine personal biases and positionality. Data interpretations were grounded in participants' words, ensuring transparency in how conclusions were reached.
- Transferability was facilitated by providing rich, contextualised descriptions of participants, settings, and findings. This enables readers to assess the relevance of the findings to other settings or populations.

These strategies collectively enhanced the trustworthiness and quality of the study's findings.

In keeping with Braun and Clarke's (2019) principles of reflexive thematic analysis, we engaged in ongoing reflexive practice throughout the research process. This study was conducted by two white Irish gay men with longstanding personal and professional engagement in LGBTQ+ health. One author is a clinician working in sexual health services, and the other is an academic whose research focuses on LGBTQ+ health inequalities and inclusive education. Our shared identities and commitments positioned us as cultural insiders with both lived and professional insight into the issues explored.

From a reflexive thematic analysis perspective, we embraced subjectivity as a resource rather than a threat to validity (Braun and Clarke 2019). We acknowledge that our positionality shaped the questions we asked, the analytic lenses we applied, and the meanings we constructed. Throughout the research process, we remained critically aware of how our experiences and assumptions could influence interpretation. Reflexive journaling, analytic memos, and peer discussions were used to surface and interrogate these influences. This reflexive stance supported transparency and depth in the development of themes.

## 3 Results

Four themes were generated from the data: Culturally aware healthcare professionals, Access and information, Specialist vs. universal services, Mental health support.

### 3.1 Theme 1: Culturally Aware Healthcare Professionals

Participants consistently identified the cultural awareness, or lack thereof, of healthcare professionals as a defining factor in their healthcare experiences. Across groups, participants described how affirming interactions with providers contributed to trust and comfort, while misgendering, heteronormative assumptions, and inappropriate questioning eroded confidence in the system. These encounters were not perceived as isolated incidents, but as indicative of systemic gaps in provider education and clinical communication. Many participants felt burdened by the need to educate clinicians about their identities or health needs, reflecting a healthcare environment that still centres cisgender and heterosexual norms. The theme highlights both the emotional labour involved in navigating these dynamics and the urgent call for comprehensive LGBT+ training in healthcare curricula and clinical practice.

Participants highlighted the critical importance of culturally aware healthcare professionals, with many sharing personal accounts of negative experiences that underscored the widespread lack of cultural competence in Irish healthcare settings. A recurring issue was misgendering, where healthcare providers failed to use participants' correct names or pronouns, leaving them feeling invalidated and disrespected.

The doctor kept calling me ‘Miss’ even though I told them I was a trans man. It felt like they weren't even listening to me. (TGD)

These experiences were described as distressing, creating barriers to seeking care and undermining trust in the healthcare system.

My first GP in the new county was predominantly to test bloods for them, but I also needed an IUD replaced and she did that, but I had to come out to her to explain vaginal atrophy and high T levels and everything. She found every opportunity to refer to me as a mother (I have no children), a wife (I was not married), and a young woman, even though I reminded her during every single appointment that I was not a woman. It just doesn't give you faith (TGD)

Another concern was the prevalence of inappropriate or sexualised questions or remarks, particularly when participants disclosed their sexual orientation or gender identity. Some felt these questions were irrelevant to the reason for their consultation and bordered on invasive curiosity rather than clinical necessity.

I went in for a sore throat, and somehow it turned into a conversation about how many sexual partners I've had. It was humiliating. (GBMSM)

It's such a dehumanising system that you get asked about masturbation when your healthcare needs have nothing to do with (TGD)

One smear test was incredibly uncomfortable afterwards when I was told I was ‘very relaxed’ in a suspicious tone. It's microaggressions like these that make receiving gender‐specific care harder. (LBWSW)

Participants also noted the pervasive presumption of heterosexuality among healthcare providers, which required them to ‘come out’ repeatedly in clinical interactions.

Every time I see a new GP, they assume I'm straight. I have to come out over and over just to make sure they don't give me advice that doesn't apply to me. (LBWSW)

This assumption, participants explained, often led to inappropriate medical advice and discomfort, further highlighting the need for more inclusive practices and awareness in clinical settings.

Having to do a pregnancy test when there is no possible way I could be pregnant because I've never had sex with a man (LBWSW)

In addition to issues related to gender and sexual orientation, participants reported bias linked to socioeconomic and racial identities. Those reliant on medical cards—indicative of lower income—described being treated dismissively by healthcare professionals. As one participant noted,

When they realised I was on a medical card, their tone completely changed. I felt like I wasn't worth their time anymore. [TGD]

As a Black queer person, I feel like I'm treated differently. They assume I don't understand the system or that I'm less educated. It's exhausting. [GBMSM]

Conversely there were also examples given of positive and affirmative experiences of healthcare.

My current GP told me he doesn't know anything about trans people but then thanked me for sharing that with him and immediately changed my name in his system even though I haven't changed it legally yet. (TGD)

What I really love about [names health service] is that there is no explaining needed, they're never shocked, they just have a nice way of going on [GBMSM]

I told my GP I'm trans. His reaction implied a hint of surprise if I interpreted his reaction correctly. He put it in my file, and the next GP I saw at the centre I'm with seemed to have seen that and used he/him pronouns. (TGD)

Participants called for comprehensive training for healthcare professionals to address biases, improve understanding of diverse identities, and foster an environment where all individuals feel respected and affirmed. Such changes, they stressed, are essential to overcoming systemic barriers and ensuring that LGBT+ individuals can access the quality care they deserve.

Healthcare workers need proper training on LGBT+ issues. It's not enough to be tolerant—they need to understand our lives and how to treat us respectfully. [GBMSM]

I shouldn't have to explain what it means to be non‐binary to my doctor. They should already know. It's exhausting to educate someone who is supposed to be helping me. [TGD]

Teach endocrinologists that progesterone is (by definition) not a progestin. Prescribe them on an informed consent basis, with a preference for progesterone due to the known risk profile of progestins. [TGD]

I also agree that older person care and cancer care providers should have extra training on LGBT+health issues and sensitivity training, along with training to proactively address and educate patients on these issues. [GBMSM]

### 3.2 Theme 2: Access and Information

Access to care and the availability of accurate, inclusive health information emerged as central concerns across all focus groups. Participants described a fragmented system in which identifying LGBT+‐competent providers required informal networks, guesswork, or online communities, often resulting in delays, stress, or avoidance of care altogether. Rather than isolated logistical issues, these barriers were perceived as structural, shaped by unequal resource distribution, digital exclusion, and the absence of institutional guidance. Participants living outside urban centres, in particular, voiced feelings of marginalisation due to geographic inequities in service provision. The data underscore how access and information function as practical necessities and as markers of institutional inclusion or exclusion within the Irish healthcare system.

Participants consistently identified access to healthcare services and the availability of accurate, inclusive information as critical barriers to achieving equitable healthcare. Many shared frustrations about the difficulty of finding healthcare providers who were knowledgeable and understanding of LGBT+ health needs. This lack of knowledge extended to both general practitioners and specialists, often leaving individuals feeling unsupported and underserved.

It feels like a lottery trying to find a doctor who actually knows about LGBT+ health—there's no centralised resource to help us identify who is safe and informed. [LBWSW]

Several participants noted that this uncertainty often discouraged them from seeking care altogether. Others spoke about relying on word‐of‐mouth recommendations within the LGBT+ community to find inclusive providers. This process, however, was described as inconsistent and time‐consuming, exacerbating feelings of marginalisation within the healthcare system.

You just end up relying on friends to tell you different things because the doctors just don't know [TGD]

The absence of a structured system for identifying LGBT+‐competent healthcare professionals emerged as a major gap in access. Many participants spoke of the need for a directory of clinicians.

It would be good to have a directory of LGBT+friendly services [LBWSW]

It's not clear whether your GP actually does things like hormones, or sexual health stuff even, if there was a centralised list then we could choose where to go and not have to spend so long figuring out [TGD]

A lack of clear and accessible information about healthcare services was also repeatedly raised as a significant barrier. Participants described the difficulty of navigating the healthcare system, particularly when seeking gender‐affirming treatments or sexual health services. For many, this process involved piecing together information from online forums, social media groups, or personal networks.

I had to rely on Facebook groups and forums to figure out how to access trans healthcare. Why isn't this information readily available? [TGD]

Those living in rural areas highlighted additional challenges, citing the lack of LGBT+‐friendly services outside major urban centres as a significant barrier. Participants described feeling excluded from the healthcare system due to their geographical location, with some even forgoing necessary care because of the travel and financial burdens involved. One participant remarked:

If you live outside Dublin, good luck trying to find anyone who understands LGBT+ health. It's like we don't exist out here. [GBMSM]

Everything is in Dublin in terms of getting some sort of awareness or services [LBWSW]

This lack of access created a stark contrast between urban and rural healthcare experiences, with rural participants feeling forgotten or marginalised. These accounts underscore the urgent need for equitable distribution of services and clear pathways for accessing care, regardless of location. Participants consistently called for better visibility of inclusive providers and centralised information systems to help navigate the healthcare landscape.

### 3.3 Theme 3: Specialist vs. Universal Services

Participants offered diverse, and at times conflicting, views on whether LGBT+ healthcare needs are best met through specialist services or integrated within universal systems. Many valued specialist services for their expertise, affirming environments, and ability to anticipate LGBT+‐specific needs without requiring disclosure or explanation. However, reliance on these services also raised concerns about accessibility, sustainability, and geographic inequity, particularly for those outside major cities. This theme reflects a broader tension between the desire for safety and competence in care, and the aspiration for an inclusive healthcare system in which all providers are adequately trained. It highlights a shared recognition that systemic change is needed, whether through expanding specialist services, integrating inclusive practices into general care, or ideally, both.

This key debate among participants revolved around the role of specialist LGBT+ services versus the integration of LGBT+ care into universal healthcare. Many participants expressed a strong preference for specialist services, citing the expertise and inclusivity they offered as essential for feeling safe and understood. These services were described as lifelines, particularly for those who had experienced discrimination or ignorance in general healthcare settings.

[Named specialist sexual health clinic] is the only place I feel like I can actually be myself and not have to explain everything about being LGBT+. They just get it. (GBMSM)

Participants highlighted that specialist services often provided a level of expertise and understanding that general practitioners and universal services lacked. For instance, clinics focused on LGBT+ health were better equipped to address specific needs, such as hormone replacement therapy for transgender individuals or sexual health for gay and bisexual men. The comfort and trust these environments fostered were described as vital for ensuring effective care.

At least when you go to [named specialist gender clinic] they at least know what to do with you, it's not perfect, but I think if it was left to GPs they wouldn't have a clue [TGD]

However, some participants raised concerns about the exclusivity of specialist services, noting that their limited availability created significant access barriers, particularly for those living outside Dublin.

If you don't live in Dublin, you're left out. Specialist services aren't accessible unless you can afford to travel, which many of us can't. [GBMSM]

This geographic disparity left participants in rural areas feeling excluded from care, with many calling for increased outreach and the expansion of specialist services beyond urban centres. At the same time, some participants questioned whether reliance on specialist services was a sustainable solution, arguing that LGBT+ healthcare should be integrated into general practice.

It shouldn't just be up to a few clinics to get it right. Every GP and hospital should know how to treat LGBT+ patients with respect. [LBWSW]

### 3.4 Theme 4: Mental Health Support

Mental health was identified across all focus groups as a critical area where LGBT+ individuals face persistent and compounding barriers. Participants described negative encounters with clinicians who lacked understanding of LGBT+ identities, with some reporting pathologising or dismissive attitudes that exacerbated rather than alleviated distress. These accounts reflected individual shortcomings and systemic failings, long waiting lists, prohibitive costs, and a lack of culturally competent services. Transgender participants, in particular, spoke of a scarcity of gender‐affirming mental health care. Collectively, these accounts highlight the cumulative toll of minority stress and the urgent need for responsive, affirming, and accessible mental health services tailored to LGBT+ lives.

Indeed, participants overwhelmingly identified mental health support as one of the most pressing areas of concern in their healthcare experiences. Many described encountering mental health professionals who lacked understanding of LGBT+ issues, leaving them feeling dismissed or misunderstood. This was particularly evident in cases where therapists or counsellors framed participants' sexual orientation or gender identity as the root cause of their mental health challenges.

I've seen therapists who just didn't understand what it means to be queer. They would focus on my sexuality as if it was the problem. [GBMSM]

Others highlighted the impact of minority stress—the chronic stress associated with discrimination and societal stigma—and noted that mental health professionals often failed to account for how these factors contributed to their mental health struggles.

I don't want to spend my therapy sessions explaining why homophobia affects my mental health. The therapist should already know this. [LBWSW]

The lack of accessible and affordable mental health services was also a recurring issue. Many participants described long waiting lists and high costs as significant barriers, with some unable to access care when they needed it most.

Mental health care is so expensive, and waiting lists are endless. By the time you get an appointment, you're already in crisis. [TGD]

Transgender participants, in particular, highlighted the difficulty of finding affirming mental health support. They described encountering professionals who either lacked knowledge of trans issues or approached their care with a pathologising lens.

Every mental health service I've tried either pathologises being trans or treats it like something they need to fix. It's exhausting. [TGD]

Participants emphasised the importance of culturally competent and affirming mental health care that recognises the unique challenges faced by LGBT+ individuals. Many called for increased training for mental health professionals to ensure they were equipped to address issues such as minority stress, internalised stigma, and the intersection of mental health with sexual and gender identity. Additionally, participants stressed the need for more accessible services, including subsidised care and shorter waiting times, to ensure timely support for those in crisis.

## 4 Discussion

Many participants in this study emphasised that their negative healthcare experiences stemmed from professionals lacking even basic knowledge of LGBT+ identities, needs, and experiences. Misgendering, assumptions of heterosexuality, and inappropriate questioning were common, underscoring the urgent need for structured, mandatory training in healthcare education. Ensuring culturally aware healthcare professionals requires comprehensive education on LGBT+ health issues throughout medical, nursing, and allied health training (McCann and Brown 2020).

These findings echo broader international research illustrating how healthcare systems often operate through heteronormative and cisnormative frameworks, where assumptions about sexuality, gender identity, and family structure are deeply embedded in clinical practices and institutional design (Zeeman et al. 2019; Hudson‐Sharp and Metcalf 2016). Participants' experiences of repeated misgendering, inappropriate questioning, or having to ‘come out’ multiple times are not isolated issues but reflective of structural norms that position cisgender heterosexual identities as defaults. Such norms are reinforced through medical curricula, intake forms, and provider‐patient interactions, and contribute to a healthcare environment where LGBT+ individuals must work to make themselves visible. These dynamics have been linked internationally to poorer health outcomes, delayed care‐seeking, and a sense of exclusion within mainstream services (Fish et al. 2021; Alencar Albuquerque et al. 2016). The persistence of these issues within Irish healthcare, despite legal advancements, signals the need for systemic cultural change, not just individual provider training, but institutional reflection and reform.

The challenges identified in this study, such as misgendering, lack of LGBT+ competent providers, and geographic inequity, closely mirror findings from UK and European contexts, where LGBT+ individuals similarly report feeling marginalised in mainstream healthcare systems (Zeeman et al. 2019; Hudson‐Sharp and Metcalf 2016). However, the Irish context presents distinct structural limitations, including a more centralised public health system and slower policy responsiveness in areas like gender‐affirming care. Despite progressive legal shifts, such as marriage equality and gender recognition legislation, healthcare policy has not kept pace. For example, Sláintecare, Ireland's 10‐year reform strategy, prioritises universal and integrated care but lacks explicit commitments to LGBT+ inclusion (Department of Health 2017). Similarly, while Ireland's National Sexual Health Strategy (2015–2020) and Mental Health Policy Sharing the Vision acknowledge LGBT+ populations, their implementation has lacked consistent funding and monitoring (Department of Health 2015; Department of Health 2020). These findings suggest that service‐level improvements must be underpinned by clear, inclusive policy directives and accountability structures to address enduring inequities in access and care quality.

LGBT+ health education should go beyond a superficial awareness of sexual and gender diversity and include in‐depth training on inclusive communication, minority stress, and the specific health disparities faced by LGBT+ individuals. The Usualising and Specificising framework (Gilmore et al. 2024) provides a structured approach for integrating LGBT+ health into healthcare education and practice, ensuring that LGBT+ identities are both routinely acknowledged (usualised) and given specific attention when needed. By embedding LGBT+ health considerations into general healthcare curricula and clinical interactions while also addressing unique needs, this approach helps to normalise inclusivity, avoiding stereotyping or fetishising; while concurrently avoiding sidelining the distinct experiences and disparities faced by LGBT+ individuals.

Participants highlighted the lack of a structured system for identifying LGBT‐competent healthcare providers, often relying on word‐of‐mouth or online forums to find safe and knowledgeable clinicians. Many called for a centralised directory of LGBT+‐friendly providers, particularly for gender‐affirming care, sexual health, and mental health. According to Henderson et al. (2018) culturally competent providers must appreciate and understand how numerous factors (including beliefs, values, and behaviours) impact a population of patients and then must tailor their care based on patients' cultures and needs. Patel and Nowaskie (2024) go on to discuss how directories of LGBTQ+ affirming clinicians must be dynamic, as what is considered competent and affirming evolves and develops. Without clear guidance on where to access competent care, however, LGBT+ individuals face additional structural barriers such as fear of discrimination that limit their ability to engage with healthcare services on an equal footing.

This lack of access created a stark contrast between urban and rural healthcare experiences, with rural participants feeling forgotten or marginalised. These accounts underscore the urgent need for equitable distribution of services and clear pathways for accessing care, regardless of location (Maria et al. 2024). The debate within focus groups around whether LGBT+ healthcare should be specialist and separate or part of integrated care underscored the need for a dual approach, combining the safety and expertise of specialist services with improved cultural competence and inclusivity in universal healthcare. While specialist services are crucial, broader systemic changes are needed to ensure that all healthcare providers can meet the needs of LGBT+ individuals (Yu et al. 2024).

The findings support growing calls for a dual approach to LGBT+ healthcare, in which specialist services continue to provide affirming, expert‐led care while mainstream services improve their inclusivity and cultural competence. Participants' preference for specialist services often stemmed from experiences of safety, affirmation, and trust, qualities not consistently found in general practice. However, specialist services are often geographically centralised and resource‐limited, creating access barriers. A more collaborative model, where mainstream providers consult with or are supported by specialist teams, could help bridge these gaps. Examples include shared care protocols, staff secondments between services, joint training initiatives, or co‐developed clinical pathways for gender‐affirming care and mental health. Such integration could improve continuity of care, reduce waiting times, and ensure that LGBT+ individuals do not have to choose between inclusivity and access. Making these collaborative mechanisms visible and standardised could also ease the burden on patients who currently must navigate fragmented systems with limited guidance.

Accounts from participants highlight the critical need for systemic reform to address gaps in mental health services in particular, to provide LGBT+ individuals with the care and support they require. Higgins et al. (2024) demonstrate that despite significant social change in Ireland over the preceding decade, mental health outcome disparities remain stark and a clear focus on LGBT+ mental health is needed.

### 4.1 Limitations

While this study provides valuable insights into LGBT+ healthcare experiences in Ireland, several limitations must be acknowledged. The sample size was relatively small, with 50 participants consenting but only 43 actively engaging. This may limit transferability, particularly for underrepresented groups such as racially diverse LGBT+ individuals, disabled people, and lower‐income populations. Additionally, self‐selection bias may have influenced the findings, as those with stronger views or prior healthcare experiences may have been more likely to participate, potentially skewing the data towards more negative experiences.

The asynchronous online focus group format provided flexibility and anonymity but may have limited the depth of discussions compared to real‐time engagement. While some participants provided longer responses to prompts, others included single words or ‘I agree’, etc. Digital literacy and internet access barriers may have excluded older participants and those in marginalised communities, affecting representation (Kelfve et al. 2020). There are limitations in the MURAL platform in terms of analytics available, so it was not possible to track numbers of logins or time spent in the focus groups, etc.

Additionally, the study offers only a snapshot in time, without longitudinal insights into how experiences evolve with policy and healthcare system changes. The lack of a structured intersectional analysis also limits understanding of how overlapping identities, such as race, disability, and socioeconomic status, shape LGBT+ healthcare experiences.

Despite these limitations, the study highlights critical barriers to equitable healthcare, underscoring the need for further inclusive, intersectional, and longitudinal research to inform policy and practice.

## 5 Conclusion

This study highlights the persistent barriers LGBT+ individuals face in accessing culturally appropriate and inclusive healthcare in Ireland. Participants' experiences underscore the urgent need for systemic change, ensuring that healthcare services are equitable, accessible, and responsive to the diverse needs of LGBT+ communities. Key issues such as cultural competence among healthcare providers, access to reliable information, equitable distribution of services, and appropriate mental health support remain significant challenges that must be addressed at both policy and practice levels. However, meaningful progress cannot be achieved without the active partnership and engagement of LGBT+ communities in shaping healthcare policies, education, and service provision (Gilmore et al. 2024).

Sustainable change requires collaborative efforts between policymakers, healthcare professionals, researchers, and LGBT+ organisations to develop inclusive, person‐centred healthcare models. Community‐led initiatives have historically played a pivotal role in driving LGBT+ health research and advocacy, and their continued involvement is essential to ensuring that policies and interventions reflect lived experiences and real needs.

Further research is needed to expand the evidence base on LGBT+ healthcare experiences in Ireland, particularly in underrepresented areas such as rural healthcare access, intersectional disparities, and long‐term health outcomes. By embedding LGBT+ health considerations into general healthcare curricula and clinical interactions while also addressing unique needs, this approach helps to normalise inclusivity, avoiding stereotyping or fetishising, while concurrently avoiding the sidelining of the distinct experiences and disparities faced by LGBT+ individuals.

As recent developments in the United States and United Kingdom demonstrate, the rights of transgender and other LGBT+ individuals remain politically contested and vulnerable to regression. These shifts serve as a stark reminder that progress in healthcare inclusion is neither guaranteed nor irreversible. In this context, the active participation of LGBT+ communities in shaping health policy and service provision is not just beneficial; it is essential for ensuring that equity gains are protected, sustained, and advanced.

## Disclosure

All data utilised in the submitted manuscript have been lawfully acquired in accordance with The Nagoya Protocol on Access to Genetic Resources and the Fair and Equitable Sharing of Benefits Arising from Their Utilisation to the Convention on Biological Diversity.

## Conflicts of Interest

The authors declare no conflicts of interest.

## Data Availability

The data that support the findings of this study are not publicly available due to privacy and confidentiality considerations but can be obtained from the corresponding author upon reasonable request.
